# The frequencies of micronuclei, nucleoplasmic bridges and nuclear buds as biomarkers of genomic instability in patients with urothelial cell carcinoma

**DOI:** 10.1038/s41598-018-35903-5

**Published:** 2018-12-14

**Authors:** Arjeta Podrimaj-Bytyqi, Ana Borovečki, Qerim Selimi, Suzana Manxhuka-Kerliu, Goneta Gashi, Isa R. Elezaj

**Affiliations:** 1 Institute of Pathology, Faculty of Medicine, University of Pristina “Hasan Prishtina”, Pristina, Kosovo; 2 Institute of Pathology “Enge”, Zürich, Switzerland; 3 School of Medicine, University of Zagreb, Zagreb, Croatia; 4 Departament of Biology, Faculty of Natural Sciences, University of Pristina “Hasan Prishtina”, Pristina, Kosovo

## Abstract

Bladder urothelial cell carcinoma (UCC) is an increasingly prevalent cancer worldwide, and thus, gaining a better understanding of its identifiable risk factors is a global priority. This study addressed this public health need with the understanding that cancer-initiating events, such as chromosome breakage, loss and rearrangement, can be reasonably used as biomarkers to evaluate an individual’s cancer risk. Overall, forty bladder cancer patients and twenty controls were evaluated for genomic instability. To the best of the investigators’ knowledge, this is the first study to perform micronucleus (MN) assays simultaneously in urothelial exfoliated cells (UEC), buccal exfoliated cells (BEC), and peripheral blood lymphocytes (PBL) in first-diagnosed, non-smoker bladder UCC patients. Additionally, the frequency of nucleoplasmic bridges (NPBs) and nuclear buds (NBUDs) in PBL was evaluated. The MN frequencies in UEC, BEC, and PBL, as well as the frequencies of NPBs and NBUDs, were significantly higher in patients than in controls. In conclusion, MN assays, particularly in UEC, may be used to identify individuals who are at high risk of developing UCC, as single or as additional triage test to UroVysion FISH test. Our results further validate the efficacy of biomarkers, such as MN, NPBs, and NBUDs, as predictors of genomic instability.

## Introduction

Bladder urothelial cell carcinoma (UCC) has become a globally common cancer, with an estimated 430000 new cases diagnosed in 2012, and it has a higher incidence rate in men than in women^1^ and increased frequency in industrial areas^2^ and in people exposed to arsenic^3^, cigarette smoking, alcohol, and so on^4^. Typically, UCC presents with haematuria, and 75% of patients with UCC initially have non-muscle invasive urothelial carcinoma (NMIUC, Ta), of low or high grade, with a high chance of recurrence (~60%) and long-term patient surveillance^5^. UCC is associated with aneuploidy (tetrasomy) of chromosomes 3, 7, and 17 as well as a loss of 9p21^6^. Cystoscopy is the gold standard for the detection of bladder tumours, followed by urine cytology and the UroVysion™ (Vysis, Downers Grove, IL) FISH test^7^. Except for cytology, other methods, although they have high detection rates, are expensive, time consuming and uncomfortable^8^.

The integrity or completeness of genomic information is a fundamental pre-requisite for life. In human tumour cells, some of one or more of the cell’s protective mechanisms are disrupted, and consequently, the cell’s genomic integrity is compromised^9^. Chromosomal instability (CIN) is a hallmark cause of the aneuploidy that is observed in most solid tumour cells and is often associated with the missegregation of chromosomes that results from improper kinetochore-microtubule attachments and the consequent presence of lagging chromosomes during anaphase^10–12^. Chromosome breakage, loss and rearrangement are important initiating events in cancer^13^, and thus, the use of biomarkers of chromosomal damage, such as micronuclei (MN), nucleoplasmic bridges (NPBs) and nuclear buds (NBUDs), to predict cancer risk and identify high-risk individuals is both valuable and imperative^9^.

MNi are defined as small chromatinic bodies that appear in the cell cytoplasm and originate from acentric chromosome fragments (particularly from chromosomes 1, 9 or 16), acentric chromatid fragments or whole chromosomes (generally sex chromosomes, especially the X chromosome) and that fail to be included in the daughter nuclei at the completion of telophase during mitosis^14^. The MN frequency appears to increase in carcinogen-exposed tissues long before any clinical symptoms are evident^15^. The MN frequency is measured by the MN assay, usually reported as the number of cells containing MNi per total cells counted. This assay was first used in exfoliated cells by Stich *et al*.^16^.

Among the changes that can occur within DNA, point mutations are the most likely to accumulate, survive, and result in tumour formation in cells that proliferate and regenerate^17^. More than 90% of cancers arise in epithelial tissues, and exfoliated epithelial cells have traditionally been used for cancer screening and monitoring by cytopathologists. These cells have a high turnover rate; within 1–3 weeks, they exfoliate along with any chromosomal damage (MN) to basal cells in their places of origin^16^. The MN assay of exfoliated cells is a cost-effective, non-invasive method that uses the formation of an MN in exfoliated cells of organs such as the oral cavity, nasal cavity, bladder, cervix, and oesophagus as an endpoint to detect endogenous, lifestyle, occupational and environmental exposures^18^.

Further application of the MN frequency to peripheral blood lymphocytes (PBL) is also used extensively in molecular epidemiology and cytogenetics^19^. The DNA damage events in PBL are specifically scored in once-divided binucleated (BN) cells that have completed nuclear division but have been blocked at the binucleated stage prior to cytokinesis. The events scored include the following: MNi (a biomarker of chromosome breakage and/or whole chromosome loss), NPBs (a biomarker of DNA misrepair and/or telomere end-fusions) and NBUDs (a biomarker of the elimination of amplified DNA and/or DNA repair complexes)^20^.

The presence of an association between MN induction and cancer development is supported by a number of observations^19,21–23^. It has been postulated that exfoliated mucosa cells have a high predictive value for the detection of carcinogenesis since the majority of human tumours are of epithelial origin^24^. Cultured cells are not needed to perform an MN assay in exfoliated cells, and its noninvasive quality makes the assay an attractive candidate for biomonitoring human populations or individuals^25^.

The aim of this study was to evaluate genomic instability in bladder UCC patients by performing MN assays in UEC and BEC and by performing a cytokinesis-block micronucleus (CBMN) cytome assay in PBL, all of which are performed simultaneously in first-diagnosed patients. Furthermore, this study will assess the frequencies of MNi, NPBs and NBUDs according to tumour muscle invasion, tumour grade, stage and recurrence.

## Results

**Figure 1:**
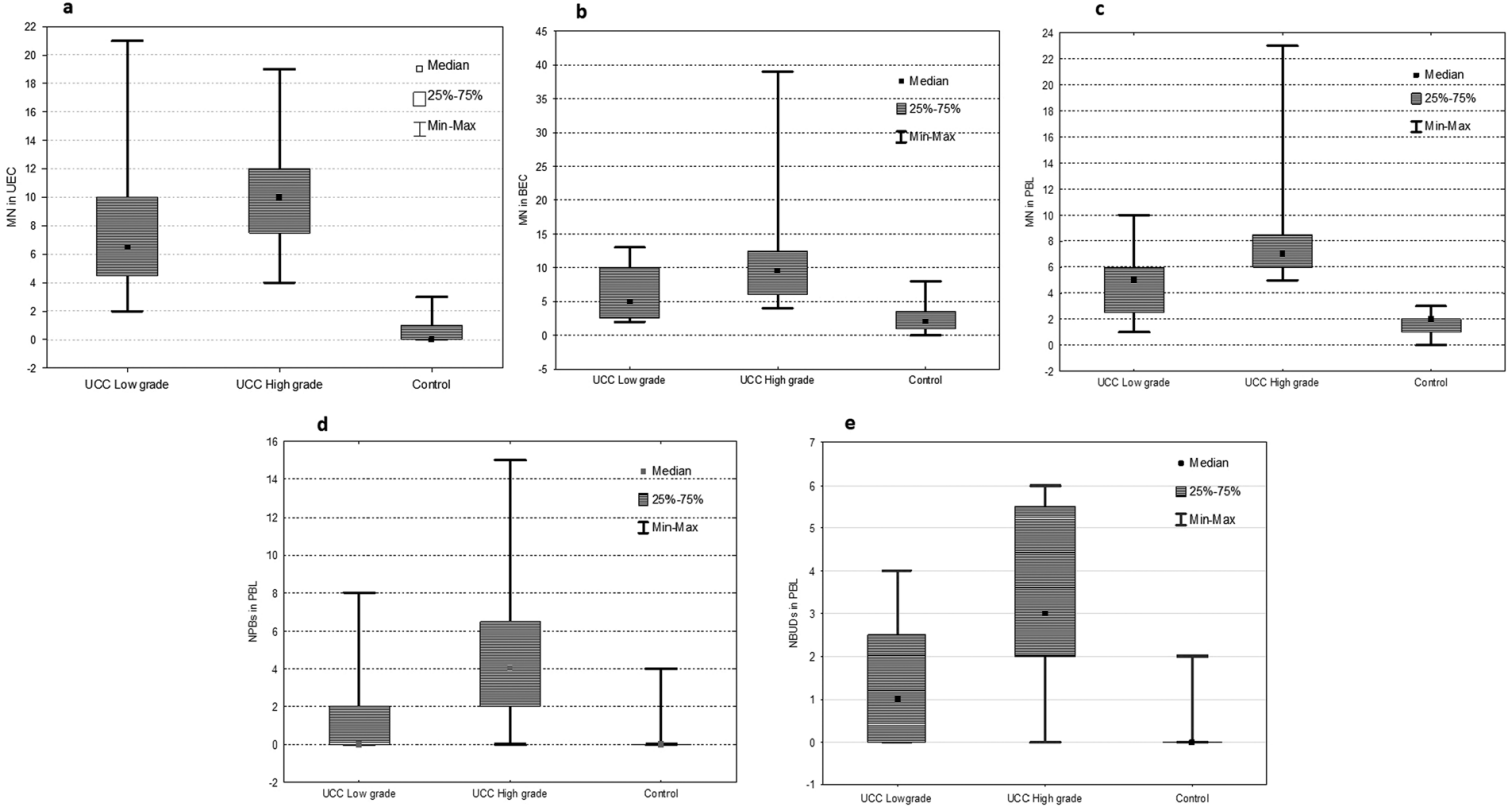
Box plots showing MN frequencies in: (**a**) UEC, (**b**) BEC and (**c**) PBL, as well as (**d**) NPB and (**e**) NBUD frequencies in PBL in the control group, low grade and high grade patient groups.

The characteristics of the study population are shown in Table 1. The MN frequencies in UEC, BEC and PBL in the patient group (n = 40) were about 17 times higher, 3 times higher, and 4 times higher, respectively, than the MN frequencies in UEC, BEC, and PBL in the control group (n = 20) (Fig. 1a–c). Furthermore, the NPB and NBUD frequencies in PBL in the patient group were 10 times higher and 12 times higher, respectively, than in the control group (Fig. 1d,e). Since study variables didn’t follow a normal distribution (tested by Kolmogorov-Smirnov test), non-parametric tests were employed to verify differences among groups: Man-Whitney U test was applied to see the differences between two independent groups, and Spearman’s correlation was applied to show the relationship between study variables.

**Table 1:** Baseline characteristics of bladder urothelial carcinoma patients and controls.

Covariates	Cases (%)	Controls
**Number**	40	20
NMIUC	16 (40%)	—
MIUC	24 (60%)	—
Age range	37–78 yrs	31–72 yrs
**Smoking status**	Non-smokers	Non-smokers
**T stage**		—
Ta	15 (37%)	—
T1	14 (35%)	—
T2	8 (20%)	—
T3	1 (2.5%)	—
T4	2 (5%)	—
Tis	—	—
Tx	—	—
Grading (WHO 2004)*		—
Low grade	20 (50%)	—
High grade	20 (50%)	—
**Recurrence**		—
Recurrent	12 (30%)	—
Non-recurrent	28 (70%)	—

Abbreviations: NMIUC –Non-muscle invasive urothelial carcinoma; MIUC- Muscle invasive urothelial carcinoma.

*For reference see: Eble JN *et al*. Pathology and genetics of tumors of the urinary system and male genital organs, (2004).

According to Man Whitney U test, statistically significant differences were found in all study variables when the patient group (n = 40) was compared to the control group (n = 20) (p < 0.001; Table 2).

**Table 2:** Mann-Whitney U test (with continuity correction) between UCC patients (n = 40) and controls (n = 20).

	Mean rank UCC	Mean rank control	U	z	p value	z adjusted	p value	Two-sided exact p value
MN in UEC	40.33	10.85	7.0	6.155	<0.001	6.202	<0.001	<0.001***
MN in BEC	38.04	15.43	98.5	4.720	<0.001	4.739	<0.001	<0.001***
MN in PBL	39.41	12.68	43.5	5.583	<0.001	5.638	<0.001	<0.001***
NPBs in PBL	36.66	18.18	153.5	3.858	<0.001	4.105	<0.001	<0.001***
NBUDs in PBL	37.50	16.50	120.0	4.383	<0.001	4.594	<0.001	<0.001***

***p < 0.001.

Spearman’s rank correlation showed no significant correlation between study variables in the control group (Table 3). However, in the patient group (Table 3), it showed: moderate positive relationships between the MN frequencies in UEC and BEC (rho = 0.427, p = 0.006); between the MN frequencies in BEC and PBL (rho = 0.382, p = 0.015), and between the MN frequency in PBL and the NPB frequency in PBL (rho = 0.327, p = 0.040). Furthermore, Spearman’s correlation showed a strong positive relationship between the frequencies of NPBs and NBUDs in PBL (rho = 0.701 and p < 0.001). There was a positive increasing trend for the relationship between the MN and NBUD frequencies in PBL, but it did not reach the level of statistical significance (Table 3).

**Table 3:** Spearman’s correlation of the MN frequencies in UEC, BEC and PBL and the NPB and NBUD frequencies in PBL in the control group, all cases group, the NMIUC and MIUC subgroups and the low and high grade subgroups.

		MN in UEC: MN in BEC	MN in UEC: MN in PBL	MN in BEC: MN in PBL	MN in PBL: NPB	MN in PBL: NBUD	NPB: NBUD
Control group (n = 20)	rho *p*	−0.091 0.702	0.082 0.732	0.264 0.261	0.049 0.831	0.049 0.837	−0175 0.459
Patients (n = 40)	rho *p*	0.427* 0.006	0.197 0.224	0.382* 0.015	0.327* 0.040	0.271 0.091	0.701* 0.000
NMIUC (n = 16)	rho *p*	0.421 0.104	0.018 0.947	0.245 0.361	−0.093 0.731	−0.194 0.472	0.861* 0.000
MIUC (n = 24)	rho *p*	0.343 0.100	0.141 0.511	0.478* 0.018	0.552* 0.005	0.450* 0.027	0.622* 0.001
Low grade (n = 20)	rho p	0.468* 0.038	0.277 0.236	0.328 0.158	0.039 0.871	0.055 0.818	0.579* 0.007
High grade (n = 20)	rho *p*	0.213 0.367	−0.163 0.492	0.339 0.144	0.185 0.435	−0.180 0.447	0.710* 0.000

^*^Statistically significant positive correlation.

Statistically significant differences were found in all study variables when patients, stratified by tumour grade into the low grade (n = 20) and high grade (n = 20) subgroups, were compared between one another (p < 0.05; Table 4). Different results were observed when patient subgroups, stratified by tumour muscle invasion into non-muscle invasive urothelial carcinoma (NMIUC, n = 16) and muscle invasive urothelial carcinoma (MIUC, n = 24), were compared: only MN frequencies in UEC were statistically significant (p < 0.044) between these subgroups, whereas MN frequencies in BEC, PBL, as well as NPB and NBUD frequencies in PBL didn’t reach the level of statistical significance (p > 0.05 in all cases, Table 5).

**Table 4:** Mann-Whitney U test (with continuity correction) between UCC low grade (n = 20) and UCC high grade (n = 20) patients.

	Mean rank Low grade	Mean rank High grade	U	z	p value	z adjusted	p value	Two-sided exact p value
MN in UEC	16.88	24.13	127.5	−1.948	0.051	−1.954	0.051	0.049*
MN in BEC	15.65	25.35	103.0	−2.610	0.009	−2.620	0.009	0.008**
MN in PBL	13.98	27.03	69.5	−3.517	0.000	−3.554	0.000	<0.001***
NPBs in PBL	14.58	26.43	81.5	−3.192	0.001	−3.258	0.001	0.001**
NBUDs in PBL	15.00	26.00	90.0	−2.962	0.003	−3.010	0.003	0.002**

*p < 0.05; **p < 0.01; ***p < 0.001.

**Table 5:** Mann-Whitney U test (with continuity correction) between subgroups stratified according to tumour muscle invasion: MIUC (n = 24) and NMIUC (n = 16).

	Mean rank MIUC	Mean rank NMIUC	U	z	p value	z adjusted	p value	Two-sided exact p value
MN in UEC	23.52	15.97	119.5	1.988	0.047	1.994	0.046	0.044*
MN in BEC	22.15	18.03	152.5	1.077	0.282	1.081	0.280	0.279
MN in PBL	22.44	17.59	145.5	1.270	0.204	1.283	0.199	0.202
NPBs in PBL	21.42	19.13	170.0	0.594	0.553	0.606	0.545	0.557
NBUDs in PBL	22.17	18.00	152.0	1.091	0.275	1.108	0.268	0.279

*p < 0.05.

Within the MIUC subgroup, Spearman’s rank correlation showed a greater correlation between study variables than it did in other subgroups. In the MIUC subgroup (Table 3), a moderate positive relationship was found between the MN frequencies in BEC and PBL (rho = 0.478, p < 0.018); between the MN frequency in PBL and NPB frequency in PBL (rho = 0.552, p < 0.005); and between the frequencies of MN and NBUD in PBL (rho = 0.450, p < 0.027), whereas a strong positive relationship was found between the NPB and NBUD frequencies in PBL (rho = 0.622, p < 0.001). Further, in the NMIUC subgroup a strong positive correlation was found only between the NPB and NBUD frequencies in PBL (rho = 0.861, p < 0.000), whereas between other variables no significant correlations were found (Table 3). According to the tumour grade, within the low grade subgroup a positive correlation was found between the MN frequencies in UEC and BEC (rho = 0.468, p < 0.038), as well as between the NPB and NBUD frequencies in PBL within the low grade subgroup and the high grade subgroup (rho = 0.579, p < 0.007; rho = 0.710, p < 0.000, respectively; Table 3).

Finally, when MNi in UEC, BEC and PBL, as well as NPBs and NBUDs, were correlated to the tumour stage and the tumour recurrences, we found that among all of these variables, only the MN frequency in PBL showed a positive trend of correlation with the tumour stage (rho = 0.276, p < 0.085), but it did not reach the level of statistical significance (data not shown).

## Discussion

It is well established that cancer has a genetic basis. Cancer development is a multistep process that involves the progressive accumulation of DNA damage, leading to the activation of oncogenes and to the loss of tumour suppressor functions, which in turn, result in changes in cell function and in the transformation of cells into malignant cells^26^. The search for cytogenetic biomarkers for the identification of groups and individuals who are at high risk of developing cancer is an important initiative in public health. By identifying and validating the markers for cancer risk, the global community can improve disease outcomes and increase the frequency of early diagnoses. In this research project, we used the MN assay in UEC and BEC and the CBMN cytome assay in PBL to evaluate genomic instability in bladder UCC patients. To the best of our knowledge, this is the first study performed on all three cell types of first-diagnosed non-smoker bladder cancer patients.

Two previous studies performed MN assays in three types of cells (UEC, BEC and PBL) to evaluate those cells in individuals exposed to arsenic exposure^27^ and cigarette consumption^28^, but none in cancer patients. These studies reported a slight increase in the prevalence of micronuclei in lymphocytes compared to exfoliated epithelial cells. However, those results may be confounded by the discrepancy in the turnover rates of these two cell types, with lymphocytes exhibiting a longer lifespan than epithelial cells^29^. In our observation, different MN means were found among the three cell types in patients with developed UCC. Specifically, exfoliated cells (UEC and BEC) had a greater prevalence of MN than PBL. Recently, there has been a growing emphasis on the value of utilizing the MN assay in exfoliated cells as a method for the early detection and/or monitoring of cancer patients, as well as the evaluation of different exposures, due to the method’s cost effectiveness and non-invasiveness^30,31^.

Given that epithelial cells are highly proliferative and that they represent the origin of more than 90% of all human cancers^32^, it is evident that epithelial cells represent a reasonable alternative to PBL for biomonitoring studies. Furthermore, while CBMN in PBL requires cell culturing, isolation of UEC can be accomplished via urine sample centrifugation alone. In the approximately thirty years since epithelial cells from human urine were first used for MN analyses, approximately 56 studies have been published^30^, most of which were performed to predict the risk of UCC based on the presence of MN-promoting genotoxic metabolites in urothelial cells.

Our results show that the MN frequency in UEC is significantly higher in patients with UCC than in individuals in the control group as well as between subgroups according to tumour grade and the tumour muscle invasion. Several confounding factors (lifestyle habits (smoking, alcohol, etc.), age, exposure to mutagen agents) may modulate MN formation and frequency^33^, but the abovementioned association in our study was independent of these factors, suggesting that endogenous factors are likely to be the most relevant causative factors that contribute to DNA damage in UCC. This result agrees with a previous study that phenotypically profiled urothelial cells in patients with a prior history of UCC^34^. Similarly, the results of a separate previous study indicated that the MN frequency in urothelial cells from bladder washings of patients with a history of UCC was higher than that of the control group^35^. Furthermore, in a retrospective study of urine samples, slides that had been identified as atypical urothelial cells and subsequently diagnosed as UCC positive were found to be positive for MN. By contrast, slides from the control group did not contain MN^36^. Based on our findings and those of these aforementioned studies, it can be concluded that subjects who are at high risk for UCC, who have UCC, or who have a history of UCC harbour and accumulate genetically unstable cells in the bladder urothelium, which may represent early precursors of new UCC or subclones from previous UCC^35^. Lastly, it can be deduced that these changes can be readily detected via an MN assay performed on urine.

Use of the MN assay in buccal cells is a well-established and standardized method^37^ that has been widely used in biomonitoring processes. In the present study, observation of the MN frequency in buccal mucosa cells revealed significantly higher levels of genomic instability in patients with UCC than in controls, similar to what was found in PBL and UEC. The MN frequency in BEC was found to have a statistically significant positive correlation with UEC and PBL in the patient group (p < 0.006, p < 0.015, respectively; Table 3). While there is a lack of literature studying the MN frequency in BEC among bladder cancer patients, a number of papers have explored the MN frequency in BEC among patients with head and neck cancer^9^, oral cancer^38^, breast cancer, and uterine cancer^39^, as well as among those with cervical pre-cancer and cancer^40,41^. These studies are consistent with our findings, showing that the MN index in PBL is significantly reflected by the MN assay in oral exfoliated epithelial cells.

The CBMN assay is a genotoxicity assay (methodology) that provides information on a variety of endpoints that reflect chromosomal breakage and rearrangements as well as gene amplification^21^. Measurement of the MN frequency in PBL is extensively used in molecular epidemiology and cytogenetics to evaluate the presence and extent of chromosomal damage in human populations exposed to genotoxic agents^22^. PBL circulate and accumulate DNA damage over their lifespans due to their close contact with different tissue microenvironments or tumour-derived substances. Thus, chromosomal aberrations in PBL are considered to be valuable biomarkers of genomic instability and predictors of cancer risk^42^.

The association between the MN frequency in PBL and cancer risk is supported by a number of observations^19,23^. The HUMN International Collaborative Project suggested that increased MN formation is associated with early events in carcinogenesis^19^.

In this study, the MN frequency in PBL in UCC patients was significantly higher than that in healthy subjects, confirming the results of Pardini *et al*., who found a higher frequency of MN in PBL and an increased frequency of NPBs and NBUDs in patients than in controls. Moreover, they found a statistically significant difference between non-muscle invasive UCC patients with controls but not between controls and muscle invasive UCC patients nor among tumour grades and recurrence^23^. In contrast, the present study found statistically significant differences in the MN, NPB and NBUD frequencies in PBL when comparing the two subgroups (according to tumour grade) between one another, but not when these variables were compared between subgroups according to tumour muscle invasion.

An increased MN frequency may be considered to be a biomarker of chromosome loss and/or breakage, whereas other anomalies, such as NBUDs, are biomarkers of gene amplification and/or the removal of unresolved DNA repair complexes, and NPBs are biomarkers of DNA misrepair and/or telomere end-fusions^43^. The increased nuclear anomalies in PBL and BEC indicate that the induction of chromosomal damage employs similar mechanisms in different tissues and that the levels of DNA damage measured in surrogate tissues may reflect those present in cancer-prone tissues^43,44^. It is estimated that each human cell is subjected to 70000 lesions per day^45^ as a result of exogenous and endogenous factors. Because the majority of DNA repair pathways are eliminated, these important pathways can be inactivated due to the action of such factors, leading to genomic instability, which in turn causes an increase in the gene mutation rate at other genomic sites and leads to cellular transformations^46^. Furthermore, abnormal active tumour substances leak from cell membranes and may enter blood circulation and interact with other tissues, destabilizing the genome.

The increased MN frequency in the PBL of UCC cases compared with that of controls could be interpreted as an altered status of the DNA damage repair system or to reflect an unknown past exposure^23^. Although our patients were non-smokers and non-alcohol consumers and they denied any professional exposure, we cannot rule out other unknown confounding factors that might have an impact on their genomic instability. There are contradictory end points after a number of investigations performed in NATO soldiers who served in the Kosovo war regarding the effect of the weapons of war, especially those with depleted uranium, on cancer risk elevation. A Norwegian investigation showed a 5-fold increase in the incidence of bladder cancer among soldiers who served in Kosovo compared with the general Norwegian population^47^. Other studies show an increased number of cancer and MN frequencies in the Bosnian population in regions exposed to depleted uranium during the Bosnian war^48,49^. In Kosovo, there is no investigation that shows the level of contamination with such agents, so this hypothesis remains at the level of speculation and should be investigated in the future.

Chromosomal instability correlates with tumour metastasis, but it remains unclear whether it is merely a bystander or a driver of metastatic progression^50^. In our study, the nonsignificant differences in variables (except for UEC) between subgroups (NMIUC and MIUC) reveal that the invasive feature of the tumour is likely not directly linked with the level of CIN. It is known that higher tumour grades harbour higher levels of CIN, but it is also known that low-grade and high-grade UCC can both be invasive. Therefore, there may be other mechanisms that generate invasive clones within UCC^51,52^ in a persistent established state of CIN.

Our data indicate that in UCC, a systemic genomic destabilization occurs and that it can be easily detected using the MN assay in UEC, BEC and PBL, which means that these cells reflect the CIN in the UCC state.

Bladder cancer continues to be challenging to detect and treat. Early diagnosis, prior to muscle invasion, can greatly impact the clinical outcome, whether measured by disease-specific survival, morbidity, or quality-of-life variables^53^. While cystoscopy and cytology are established standards, the UroVysion™ FISH test is a new but expensive and technically demanding approach that focuses on chromosomal aneuploidy using fluorescent *in situ* hybridization (FISH)^54^ and is mainly used as a surveillance test for NMIUC patients.

It is well recognized that high pre-diagnostic levels of MN are associated with an elevated risk for cancer development. It is also known that tetrasomy of chromosomes 3, 7 and 17 and loss of 9p21 can be detected in urine samples 1–3 years before diagnosis^11^. Although more extensive studies are needed, we recommend the following practical approach for cancer screening: combined testing for bladder cancer, in which the MN assay in UEC might serve as a triage-test for the identification of people who are at high risk for bladder cancer so that they can be referred for UroVysion™ FISH testing. This method could reduce the number of unnecessary tests.

In conclusion, our data show that genomic instability can be detected through the MN assay in target cells (UEC) as well as in surrogate cells (BEC and PBL). The extent of DNA damage varies among the three cell types, but their simultaneous appearance suggests that similar mechanisms can cause a systemic genomic instability in different types of cells during carcinogenesis. Although further studies are needed for standardization, our findings support the data in the field that indicate that the MN frequency in exfoliated cells (especially in UEC) may be a potential biomarker for the early detection of bladder cancer and for patient surveillance. The MN frequency in exfoliated cells might also be able to be used as an additional triage test prior to the UroVysion™ FISH test.

### Strengths and limitations

This study used a very homogenous sample. The inclusion criteria for eligibility, especially the non-smoking status, made the sample collection phase to be a long and a challenging process, that resulted in a relatively small sample size. Despite this, the study is valuable as an explorative one, significantly pointing the presence of a systemic genomic instability due to bladder cancer disease. Although, further studies are needed to set up or to standardize MN frequencies in UEC, BEC, PBL as well as NPBs and NBUDs in PBL, to be routinely included as predictive diagnostic criteria for bladder cancer early diagnosis, and cancer surveillance.

## Subjects and Methods

### Subjects

The study sample consisted of 60 male participants. Subjects were divided into two groups as follows:

1. Patient group – 40 patients diagnosed with bladder UCC.
2. Control group - 20 healthy men.

This study was conducted at the Urology Clinic and Institute of Pathology of the University Clinical Center of Kosovo (UCCK) and Department of Biology, Faculty of Natural Sciences, University of Pristina “Hasan Prishtina”. The investigated patients were collected prospectively from 2014 to 2017. The age range of the patient group was 37 to 78, while the controls were between 31 and 72 years of age.

To be chosen for this study, each prospective subject was required to conform to the following criteria: non-smoker, non-alcohol consumer, and no history of chemo- or radiotherapy, as confounding factors^4,55–58^.

Patients were first verbally briefed regarding the purposes of the study and were introduced to the sample collection procedure. Afterwards, patients were asked to provide information regarding their relevant personal information (name, age, occupation), family data (diseases with familiar predispositions) and medical data (present diseases, past diseases, habits). Informed written consent for sample collection was also obtained from each participant. The following three tissue samples were taken from all participants prior to the operation and diagnosis: urine, buccal mucosa exfoliated cells and blood.

This research was approved by the Scientific Ethical Committee of UCCK (Nr. 566, date: 7 February 2012). All methods were performed in accordance with the relevant guidelines and regulations.

### Methods

#### MN assay in UEC

Midstream urine of the second or third void of the day was requested from each participant. The urine was collected in sterile urine containers and was processed within two hours. The volume of the voided urine ranged from 150–200 ml.

Urine samples were transferred to centrifuge tubes and centrifuged at 2000 rpm for 15 min. The supernatant was then discarded without disturbing the pellets of urothelial cells. The pellets were washed twice with 0.9% NaCl and centrifuged. Cell suspensions were spread onto clean, preheated (40 °C) glass slides and allowed to air dry for 5–10 min. The slides were fixed in Carnoy I fixative (methanol: glacial acetic acid, 3:1) at 0 °C for 20 min and air dried. The slides were stained with May-Grunwald’s stain (0.25%) for 5 min, counterstained with Giemsa (4%) for 10 min and mounted in DPX. Four slides were prepared for each individual.

**Figure 2:**
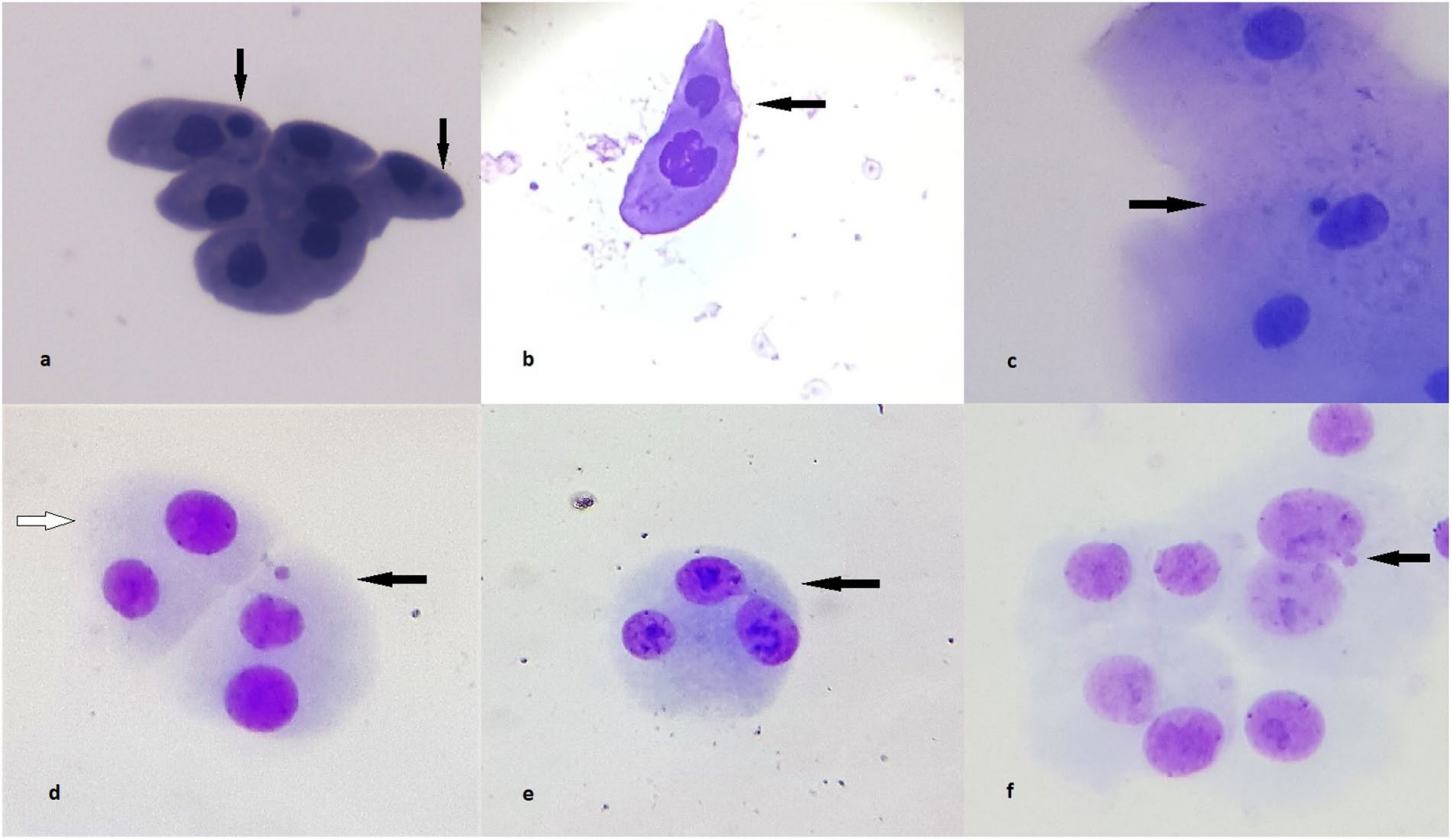
Representative images showing nuclear anomalies observed in the three types of cells: (**a**,**b**). micronuclei in urothelial cells (arrows), (**c**) micronucleus in buccal cells (arrow), (**d**) normal binuclear lymphocyte (empty arrow); binuclear lymphocyte with a micronucleus (arrow), (**e**) nucleoplasmic bridge in peripheral blood lymphocytes (arrow), (**f**) nuclear bud in peripheral blood lymphocytes, (magnification x400).

Following the method of Reali *et al*.^59^ and Fortin *et al*.^60^, at least 1,000 urothelial cells per individual were analysed under a light microscope and confirmed by a second observer (Fig. 2a,b).

#### MN assay in BEC

Buccal exfoliated cells were prepared and analysed after the method of Thomas *et al*.^37^ and Tolbert *et al*.^61^. First, subjects removed unwanted debris from their oral cavities using a distilled water rinse. BEC were then obtained by rolling a cytobrush against the buccal mucosa ten times in a circular motion. Next, the heads of the brushes were individually placed into separate 30 ml yellow-top containers, each of which contained a mixture of the following components: buccal cell buffer (0.01 M Tris-HCl; Sigma T-3253), 0.1 M EDTA tetrasodium salt (Sigma E5391), and 0.02 sodium chloride (Sigma S5886) at pH 7.0. The containers were then agitated to disperse cells. Cells were transferred into separate TV-10 centrifuge tubes and centrifuged for 10 min at 1500 rpm (MSE Mistral 2000). Slides containing two spots of cells were air dried for 10 min and then fixed in ethanol:acetic acid (3:1) for 10 min. Slides were then air dried for 10 min prior to staining with Giemsa (5%). At least four slides were prepared per individual, and 2,000 cells were analysed per case. The results are expressed as the number of micronucleated cells per 1000 counted cells (‰).

All the scoring criteria for observing these nuclear anomalies (Fig. 2c) were taken from the corrected version of the Protocol of Thomas and Fenech^37^.

#### CBMN cytome assay in PBL

Blood samples (5 ml) were collected via venipuncture into vacutainer blood tubes containing a lithium heparin anticoagulant (BD Plymouth. Pl6 7BP. UK). Whole heparinized blood (0.5 ml) was added to 5 ml of complete PB-Max Karyotyping medium for cell cultivation (Invitrogen, California, USA). All cultures were duplicated and incubated at 37 °C for up to 72 hours. Cytochalasin B (Sigma, St Louis, MO, USA) at a final concentration of 4 μg/mL was added 44 hours after the incubation was initiated. The cells were centrifuged (Sigma, Germany) at 800 rpm for 10 min and treated with a hypotonic solution (0.075 M KCl). The cell suspension was then fixed in Carnoy I fixative (methanol:acetic acid, 3:1) three times and centrifuged (300 g/8 min) after each fixation. Finally, the centrifuged cells were resuspended in a small volume of fixative and spread onto specially prepared, cold, and lamp-dried slides. The slides were stained with Giemsa solution (5%). At least six slides per individual were prepared. Finally, using a light microscope, the MNi, NPBs and NBUDs in PBL (Fig. 2d–f) were scored independently by two scorers for 1000 binucleated cells (500 per culture) according to the method of Fenech^20^.

Microscopic analyses and photography were performed with an Olympus CX43 light microscope with a Moticam 10^+^, 10.0 MP camera.

#### Statistical analysis

Since the study variables (the frequency of MN in UEC, BEC and PBL as well as NBUD and NPB in PBL) did not follow normal distribution (tested by Kolmogorov-Smirnov test), non-parametric methods were used. Mann-Whitney U test was applied for comparison of two independent groups. Correlation analysis between study variables were calculated as Spearman’s rank correlation coefficients. All the analyses were performed by Statistica (data analysis software system), version 13 (TIBCO Software Inc. (2018). The results were expressed as mean ± standard deviation (SD). A value of p < 0.05 was considered statistically significant.

## Data Availability

All data generated or analysed during this study are included in this manuscript.
